# Tag mechanism as a strategy for the RNA replicase to resist parasites in the RNA world

**DOI:** 10.1371/journal.pone.0172702

**Published:** 2017-03-02

**Authors:** Sanmao Wu, Chunwu Yu, Wentao Zhang, Shaolin Yin, Yong Chen, Yu Feng, Wentao Ma

**Affiliations:** 1 College of Life Sciences, Wuhan University, Wuhan, P.R.China; 2 College of Computer Sciences, Wuhan University, Wuhan, P.R.China; Max-Planck-Institut fur terrestrische Mikrobiologie, GERMANY

## Abstract

The idea that life may have started with an “RNA world” is attractive. Wherein, a crucial event (perhaps at the very beginning of the scenario) should have been the emergence of a ribozyme that catalyzes its own replication, i.e., an RNA replicase. Although now there is experimental evidence supporting the chemical feasibility of such a ribozyme, the evolutionary dynamics of how the replicase could overcome the “parasite” problem (because other RNAs may also exploit this ribozyme) and thrive, as described in the scenario, remains unclear. It has been suggested that spatial limitation may have been important for the replicase to confront parasites. However, more studies showed that such a mechanism is not sufficient when this ribozyme’s altruistic trait is taken into full consideration. “Tag mechanism”, which means labeling the replicase with a short subsequence for recognition in replication, may be a further mechanism supporting the thriving of the replicase. However, because parasites may also “equip” themselves with the tag, it is far from clear whether the tag mechanism could take effect. Here, we conducted a computer simulation using a Monte-Carlo model to study the evolutionary dynamics surrounding the development of a tag-driven (polymerase-type) RNA replicase in the RNA world. We concluded that (1) with the tag mechanism the replicase could resist the parasites and become prosperous, (2) the main underlying reason should be that the parasitic molecules, especially those strong parasites, are more difficult to appear in the tag-driven system, and (3) the tag mechanism has a synergic effect with the spatial limitation mechanism–while the former provides “time” for the replicase to escape from parasites, the latter provides “space” for the replicase to escape. Notably, tags may readily serve as “control handles”, and once the tag mechanism was exploited, the evolution towards complex life may have been much easier.

## Introduction

The idea that an RNA world [1] existed before the DNA and protein-based life has become a central hypothesis in regard of early evolution [2–5]. Moreover, it has been argued that the RNA world may have represented the start point of life, as described in the so-called “RNA first” scenario [6,7]. In this scenario, it has been popularly assumed that an RNA replicase, namely, a template-dependent RNA synthetase ribozyme capable of catalyzing its own replication (via an intermediate of its complement), should have emerged first [5–8]. If this RNA species, benefiting from its own function, could indeed spread (through replication) in the prebiotic environment, then its emergence may have represented a significant event in the origin of life, marking the outset of Darwinian evolution [9].

There have been long-standing efforts to construct such an RNA replicase by in vitro evolution experiments [10–15]. Recently, an artificial ribozyme was reported being able to catalyze the copying of some RNA templates as long as itself (~200nt) [16], and it seems that the chemical potential of RNA to fulfill its own replication has almost been confirmed. Moreover, an interesting study demonstrated that such an RNA polymerase ribozyme may appear by assembling from simple RNA oligomers (e.g., about 30nt long, possibly arising from prebiotic chemistry) under certain conditions involving freeze-thaw cycles [17]. However, even if such an RNA replicase is chemically feasible and may appear (as one or several molecules) in the early RNA world as well, how could the ribozyme be selfish enough to favor its own replication, while ignoring other RNA molecules under the same circumstances [6,7], and thus spread (thrive) in the system? None of these experimental studies could offer an answer. This problem, which is referred to as the “parasite” problem, is not a chemical problem, but a problem of evolutionary dynamics [9].

To date, theoretical modeling and computer simulation has become a most powerful approach to address issues concerning evolutionary dynamics in the field of origin of life [9,18–21]. In fact, by computer simulation, it has been shown that spatial limitation [22] may have played an important role for an RNA replicase to overcome the parasite problem–if the ribozyme’s molecules tend to assemble together and be kept away from parasite molecules, they may be more likely to catalyze their own replication. However, if the replication process is considered in detail, i.e., not as an instantaneous step, then the replicase may be difficult to spread in the system, even if spatial limitation is assumed [23]. The reason should be that when the replicase is functioning, it would bind on the template, and thus have less chance to work as a template itself, compared with other RNA molecules [24–26]. In other words, the replicase, which needs to be selfish for the sake of its spreading, is, in practice, altruistic. It would be difficult for this altruistic ribozyme to resist parasites–counting merely on the spatial limitation. Indeed, in a previous modeling study, we revealed that if the replicase was a template-dependent ligase instead of a template-dependent polymerase (as it is usually assumed), it may spread in the system–because the ligase would loosely bind to the template (unlike the polymerase), it may easily drop from the template and thus have more chance to act as a template itself [27]. However, obviously, the efficiency of the ligase-type replicase would be quite limited, especially considering its “loosely binding” feature. It seems that even if the first replicase had been of the ligase-type, the emergence of a polymerase-type replicase, which bound the template tightly and was more efficient, would have been unavoidable before the RNA world became advanced, with more and longer “genes” needed to be replicated.

Then, beyond spatial limitation, can the RNA replicase have other strategies that aid in overcoming the parasite problem? Indeed, a possible solution is the tag mechanism [6,7,28]. The ribozyme may recognize itself through a short subsequence. Actually, tag mechanisms are commonly used mechanism in modern life, including in the recognition of the starting sites of DNA/RNA that should be replicated, transcribed, or translated. For example, the “origin of replication” represents a specific subsequence within DNA which may be bound by special proteins that can initiate the DNA’s replication. Thus, the replication could always start at precisely the same locus, while neglecting others. As for transcription and translation, the corresponding cases are the promoter and the ribosome-binding site, respectively. In fact, such specific recognition events at the molecular level are so common in modern life that we can cite pages of examples, indicating that it is an easy-to-exploit mechanism. Thus, in the earliest scenario concerning life, the RNA polymerase ribozyme may also have made use of it–adding a specific tag sequence to the ribozyme (and its complementary chain) may represent a solution to the parasite problem. That is, through the tag, the ribozyme can recognize and replicate itself but neglect unrelated species. However, apparently, there is a new problem: the unrelated species, i.e., parasites, can also contain the tag! Moreover, as it was pointed out, with respect to the “sequence space”, the relative abundance of the replicase versus the parasites does not change at all when the tag is added [29]. In other words, the tag mechanism is “equitable” for the replicase and the parasites. In this consideration, then, it seems that the tag mechanism can hardly help to solve the parasite problem.

To study in depth whether the tag mechanism can help the RNA replicase (polymerase-type) to overcome the parasite problem, we conducted a computer simulation. The results provided a positive answer to this question. In a tag-ruled system, parasite molecules, especially those strong ones, are more difficult to appear. This effect, synergizing with the spatial limitation effect, would result in a “better” dynamic spatial distribution structure, which leads to the prosperity of the RNA replicase.

## Methods

Our computer simulation was based on a Monte-Carlo method (similar to the cellular automaton) that we established approximately a decade ago [23]. It has been used to conduct a series of studies on the evolutionary dynamics involved in the development of the RNA world [27,30–34]. In particular, the model used here is directly derived from that of a study on the behavior of a ligase-type RNA replicase in the early RNA world [27]. In the present study, our focus turns to a polymerase-type RNA replicase, which exploits the so-called tag mechanism to recognize itself and its complementary sequence during the catalysis of its own replication.

**Fig 1:**
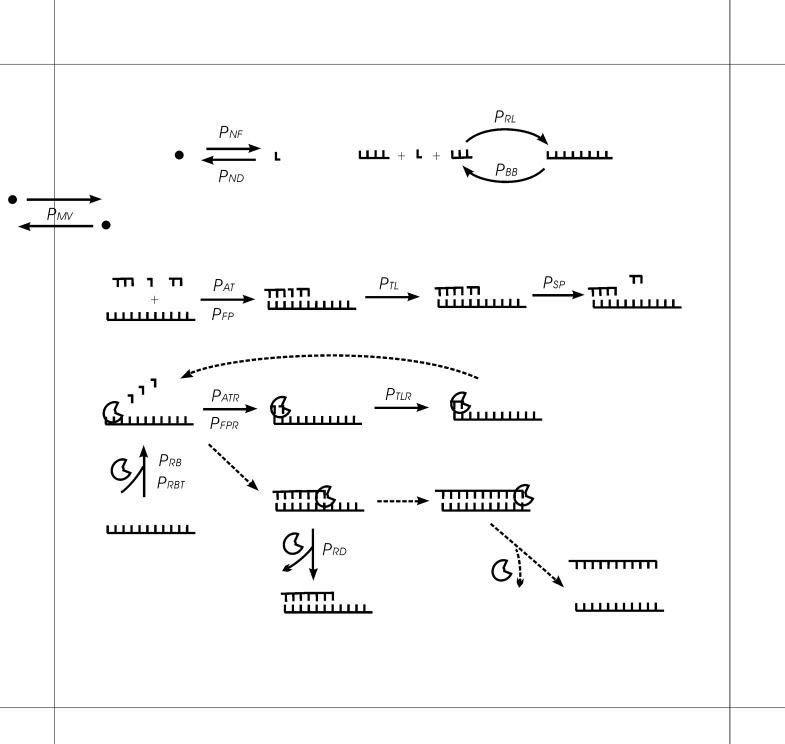
Events occurring in the system and their associated probabilities. The diagram shows these events in a background of one grid room in the *N* × *N* grid. L-shapes represent nucleotides, dots represent raw materials (nucleotide precursors) and crescent-shapes represent the replicase. Dashed lines outline a complete turn of the template-directed copying catalyzed by the polymerase-type replicase. Note that nucleotides and RNAs may also move into or out of the room, with a probability in relation to *P*_*MV*_ (see the text for details).

Generally, we assume a two-dimensional surface for the system, with an *N* × *N* square grid, among which units are distributed, including the raw materials to synthesize nucleotides (or say, nucleotide precursors, in quotient of nucleotides), nucleotides, and RNAs. An RNA containing a characteristic domain (arbitrarily presumed) is assumed to be able to act as a template-dependent polymerase ribozyme. A short specific sequence (also arbitrarily presumed) is assumed to be a tag (only valid when at the 3’-end of an RNA, see below), and its reverse complement is referred to as a “reverse-tag” (at the 5’-end of an RNA). Here, using arbitrarily presumed sequences to characterize the replicase and the tags is on account of our ignorance in this aspect and based on our understanding that the key factor in the evolutionary dynamics is the correspondence between a sequence and its relevant function rather than the actual sequence. Also based on such an understanding, we tend to adopt sequences that are much shorter than those plausible in reality, in order to avoid cumbersome computations–typically, 9nt for the characteristic domain of the ribozyme and 3nt for the tag and reverse-tag, as adopted in the cases shown here in the results. But note that a tag shorter than 3nt is obviously not representative and should be avoid. A tag should substantially label specificity–just suppose that a 2nt tag means 1/16 of random sequences would carry it, and even more extremely, a 1nt tag means 1/4 of random sequences would carry it. In a Monte-Carlo step (time step), for units in a grid room, the following events may occur (also refer to Fig 1):

1. A quotient of raw materials may form a nucleotide (randomly as A, G, C, or U) with *P*_*NF*_ (see Table 1 for a description of the abbreviation and those appearing in the following).
2. A nucleotide may decay to a quotient of raw materials with *P*_*ND*_.
3. RNAs (including nucleotides) may conduct random ligation with *P*_*RL*_, thus forming longer RNAs.
4. A polymerase ribozyme may bind onto an RNA template with *P*_*RB*_, but if the template is a single chain containing the tag at its 3’-end, then the binding probability would be *P*_*RBT*_. It should be noted that to be bound by a polymerase, the RNA template must be longer than the cover-length of the polymerase. Considering the ribozyme’s folding, the cover-length is assumed to be (L+2)^1/2^, where L is length of the characteristic domain of the ribozyme.
5. An RNA template may attract a substrate (nucleotide/oligomer) with *P*_*AT*_ if the corresponding residue(s) could base-pair correctly or not correctly but within the limitation of false-pairing *P*_*FP*_. An RNA template with a bound polymerase ribozyme may attract a substrate (nucleotide) with *P*_*ATR*_ if they could base-pair correctly or not correctly but within the limitation of false-pairing *P*_*FPR*_. The *P*_*FP*_ and *P*_*FPR*_ are associated with the fidelity of the replication.
6. Substrates assembling on an RNA template may conduct template-directed ligation with *P*_*TL*_ in a non-enzymatic mode or with *P*_*TLR*_ under the catalysis of a bound polymerase ribozyme.
7. A bound polymerase ribozyme would drop from its template when the whole chain is copied, or may drop with *P*_*RD*_ when the copying has not yet been completed.
8. An RNA double chain region may dissociate, given the separation of base pairs with *P*_*SP*_−the probability of the dissociation is actually set as *P*_*SP*_^n/2^, where n is the number of the base pairs, and the introduction of the 1/2 corresponds to the consideration that self-folding of the single chains may aid in the dissociation.
9. An RNA may degrade into shorter RNAs (including nucleotides), given the breaking of phosphodiester bonds with *P*_*BB*_. The breaking probability for the bond at the end of an RNA chain (either the 3’ or 5’-end) is *F*_*EB*_ times that for those bonds within the chain. When the breaking site is within a double-chain region, the breaking probability becomes *P*_*BB*_^2^. The bound polymerase ribozyme on the template has a chain-breaking probability of *P*_*BB*_^3/2^, because the self-folding of the ribozyme may protect it from degradation to an extent. Certainly, when the ribozyme’s chain breaks, the resulting pieces would drop from the template.
10. A unit may move to an adjacent grid room, the probabilities of which for a quotient of raw materials is *P*_*MV*_, for a nucleotide is assumed to be *P*_*MV*_/2, and for an RNA is assumed to be (*P*_*MV*_/2)/m^1/3^, where m is the mass of the RNA relative to a nucleotide. The introduction of the 1/3 corresponds to the approximation that the diffusion rate is proportional to a one-dimensional factor of that RNA molecule.

**Table 1:** Parameters used in the Monte Carlo simulation.

**Probabilities**	**Descriptions**	**Magnitudes^a^**	**Values^b^**
*P*_*AT*_	(Nts or oligomers) attracted by template (non-enzymatic)	[0.001, 0.01]	0.01
*P*_*ATR*_	(Nts) attracted by template with a ribozyme binding on	[0.1, 0.9]	0.9
*P*_*RB*_	Ribozyme binding onto template (without the tag)	[1×10^−4^, 0.01]	0.001
*P*_*RBT*_	Ribozyme binding onto template containing the tag	[0.1, 0.9]	0.9
*P*_*NF*_	Nucleotide formation.	[0.001, 0.01]	0.01
*P*_*ND*_	Nucleotide decay	[0.001, 0.01]	0.001
*P*_*RL*_	Random ligation of nucleotides and oligonucleotides	[1×10^−7^, 1×10^−5^]	2×10^−6^
*P*_*BB*_	Phosphodiester bond breaking in an RNA chain	[1×10^−6^, 1×10^−4^]	2×10^−5^
*P*_*FP*_	False base-pairing	[0.01, 0.1]	0.01
*P*_*FPR*_	False base-pairing with ribozyme on the template	[1×10^−4^, 0.01]	0.001
*P*_*TL*_	Template-directed ligation (non-enzymatic)	[1×10^−4^, 0.01]	0.001
*P*_*TLR*_	Template-directed ligation catalyzed by the ribozyme	[0.1, 0.9]	0.9
*P*_*RD*_	Ribozyme dropping from the template.	[1×10^−4^, 0.01]	0.001
*P*_*SP*_	Separation of a base pair.	[0.01, 0.1]	0.08
*P*_*MV*_	Movement	[1×10^−4^, 0.01]	0.001
**Others**	**Descriptions**	**Magnitudes**^a^	**Values**^b^
*N*	The scale of the system (i.e., it is defined as an N × N grid)	[20, 40]	20
*T*_*NPB*_	Total nucleotide precursors introduced in the beginning	[20000, 80000]	40000
*F*_*EB*_	Factor for end-breaking of an RNA chain	[10, 30]	20

a. The magnitudes represent the general scopes of the values that were adopted in our study.

b. The values correspond to those used in the case shown in Fig 2A.

For the setting of the probabilities, some principles should be followed. According to the tag mechanism, *P*_*RBT*_ should be much greater than *P*_*RB*_. Because the RNA replicase works as a polymerase ribozyme, *P*_*RD*_ should be quite small, *P*_*ATR*_>>*P*_*AT*_, *P*_*TLR*_>>*P*_*TL*_, and *P*_*FPR*_>*P*_*FP*_. Other considerations may include *P*_*TL*_ >> *P*_*RL*_, *P*_*BB*_ ≥ *P*_*RL*_, and *P*_*NF*_ ≈ *P*_*ND*_. The general magnitudes used to set the parameters in the study are listed in Table 1.

At the beginning of a simulation, raw materials are introduced into the system such that they are randomly distributed among the grid rooms. The raw materials would form nucleotides, which then form RNAs by random ligation–the system evolves step by step through the events in the model (as described above). A few molecules of the polymerase ribozyme (i.e., the RNA replicase) are inoculated soon after the initial step. The quantities of the ribozyme and the parasites are monitored by checking relevant subsequences (i.e., the ribozyme’s characteristic domain, the 3’-tag and the 5’-reverse-tag). Other features could also be traced or recorded if necessary, such as the quantities of raw materials, nucleotides, the chain-length distribution of the RNAs, and the spatial distributions of the replicase and parasites.

## Results

### Spread of the tag-driven polymerase-typed RNA replicase

The simulation showed that the tag-driven polymerase may thrive in the system. Fig 2A shows a typical case of such a situation. Initially, a few molecules of the polymerase with a tag and a reverse-tag (at its 3’- and 5’-ends, respectively) were inoculated, and then they spread in the system, reaching a level obviously higher than that of parasites. Here, a parasite refer to any RNA molecule longer than the polymerase’s cover-length (see “Methods”) that contains a tag at its 3’-end but is not a ribozyme or its complement. They are called “parasites” because they may occupy the polymerase and thus interfere with the self-favoring replication of the ribozyme. Actually, if a parasite does not contain a reverse-tag at its 5’-end, it cannot really replicate because its complementary sequence, even being copied out, would not contain a 3’-tag, and thus cannot be recognized by the polymerase. So here we refer to a parasite only containing a 3’-tag as a “pseudo-parasite”. Indeed, “true-parasites” should be those really able to replicate. Thus, here only the parasites containing both a 3’-tag and a 5’-reverse-tag can act as “true-parasites” (note that all parasites in a system without the tag mechanism are true parasites, see below). In fact, to determine whether the polymerase’s spread is caused by its own function, a control RNA species was introduced together with the ribozyme at the initial inoculation, in the same number of molecules. The control species contains both a 3’-tag and a 5’-reverse-tag, thus being a true-parasite, but it is rather “weak” because its sequence is long (the same as that of the ribozyme)–later in this paper we will address the issue of parasite length.

**Fig 2:**
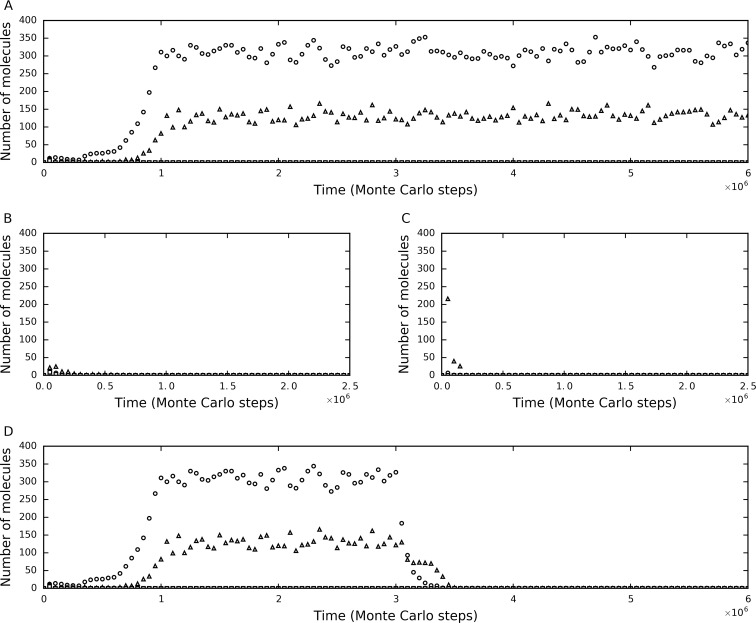
The tag mechanism is important for the polymerase-type RNA replicase to resist parasites and spread in the system. (**A**) The system in which the tag mechanism works. See Table 1 (the last column) for the parameter values adopted in this case. Characteristic domains of the polymerase: CGACGUCAG; 3’-tag: AUG; and 5’-reverse-tag: CAU. At step 1×10^4^, four grid rooms, chosen randomly, were each inoculated with five molecules of the following RNA species: a double-tagged polymerase (AUGCGACGUCAGCAU), the complement of the double-tagged polymerase, a double-tagged control (AUGCAGUCGUACCAU) and the complement of the double-tagged control. Circles represent the double-tagged polymerase plus its complement. Triangles represent the RNA species (longer than the polymerase’s cover-length) that contain a 3’-tag but not a characteristic domain of the polymerase (or its complement). Squares represent the double-tagged control plus its complement. (**B**) The system in which the tag mechanism does not work. The situation is the same as that in the case shown in A, except that *P*_*RB*_ is set to be equal to *P*_*RBT*_ (0.9). (**C**) The system in which the tag mechanism does not work and also, no tag sequences are introduced. The situation is the same as that in the case shown in B, except that the polymerase and its complement (circles), as well as the control and its complement (squares), do not contain the two tags. Triangles represent the RNA species (longer than the polymerase’s cover-length) that do not contain the characteristic domain of the polymerase (or its complement). (**D**) The same case as that shown in A, but *P*_*RB*_ is turn up to be equal to *P*_*RBT*_ (0.9) at step 3×10^6^.

However, when *P*_*RB*_ is set to the same value as that of *P*_*RBT*_, which means the tag mechanism does not work, the inoculated polymerase cannot spread (Fig 2B). It may be argued that in this case, the failure of the polymerase is caused by the useless subsequences, the tag and reverse-tag, which retarded its replication. To exclude such a potential reason, we conducted another simulation in which the inoculated polymerase does not containing the useless tag and reverse-tag, and the result showed that the polymerase could not spread either (Fig 2C). Moreover, in fact, after the replicase spread in the system, when we turned up the value of *P*_*RB*_ midway to equal that of *P*_*RBT*_, the prosperity would collapsed immediately (Fig 2D). These results are in accordance with our previous work in which a positive outcome for the thriving of a polymerase-type RNA replicase, based only on the mechanism of spatial limitation, was not found [23,27].

As mentioned in the introduction, the tag mechanism appears to be “equitable” for the replicase and the parasites. Obviously, such an “equitable” rule could result in different influences on the replicase and the parasites, leading to the prosperity of the replicase. Then why? Where does the difference come from?

### Why the tag mechanism can work

There are two ways for an RNA molecule to appear in the system, de novo or through replication. Except for in the very beginning, nearly all the replicase molecules in the system arise from replication. The introduction of the tags would add a little burden to the replication, but this is trivial (when compared with its influence on parasites, as explained below). In contrast, parasitic molecules may form de novo at any time, through various routes (see below), and this is an important way for them to appear. Additionally, because the parasites cannot replicate by themselves, they will tend to die out if the replicase in the same region dies out, unless they can move to a new region containing the replicase, which is not always successful, especially if the spatial limitation takes effect (see “Synergism between the tag mechanism and the spatial limitation mechanism” for details). That is, parasites, often, “have to” appear de novo. Notably, this is just the point at which the tag mechanism could work.

**Fig 3:**
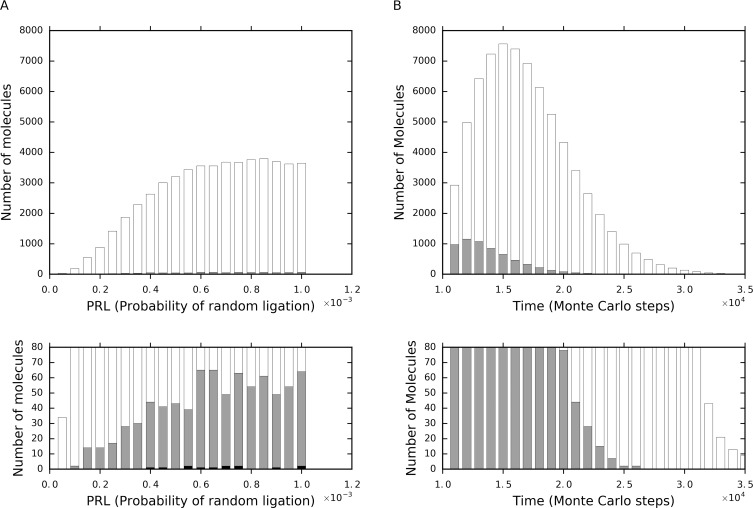
Influence of the tag mechanism on the two routes of the parasites’ de novo appearance. (**A**) Naturally appearing through random ligation. The data are from different cases with different *P*_*RL*_ values, and they were sampled at step 5 × 10^5^ in these cases, which has the same parameter settings (except *P*_*RL*_) as the case shown in Fig 2A but without the initial inoculation of the replicase (and the control). All of the RNA molecules (longer than the cover-length of the polymerase) that are not the replicase or its complement were counted. Because of the limitation of raw materials, the molecules would not increase with the enlargement of *P*_*RL*_ continuously. White bars denote the molecules without the tag, gray bars denote the 3’-tagged molecules, and black bars denote the double-tagged molecules. The lower half is an amplified version of the upper half, to show those extremely rare double-tagged parasites (black bars) clearly. (**B**) Derived from the ribozyme (here only degradation is considered, while partial replication is another important route–see the text). The data are from one case but recorded at different time steps. 10^4^ double-tagged replicase molecules were inoculated into the system at step 1 × 10^4^, and only the event of RNA degradation is allowed (*P*_*BB*_ = 2×10^−5^) in the model. The bars are interpreted the same way as in ***A***, and the lower half is an amplified version of the upper half.

The tag mechanism makes the de novo appearance of parasites more difficult. Firstly, in a system without the tag mechanism (i.e., a “tag-free” system, for short), any RNA species that appeared through the random ligation of nucleotides and oligonucleotides would act as parasites (if only they reaches the polymerase’s cover-length), and all of them would be true-parasites. In a system with the tag mechanism (i.e., a “tag-ruled” system, for short), however, only those happen to contain a 3’-tag could be parasites, and only those happen to contain both a 3’-tag and a 5’-reverse-tag could act as true-parasites! Indeed, a test simulation study with no initial inoculation (of the RNA replicase, the control and their complements) demonstrates that within the naturally formed RNA molecules (coming largely from the random ligation), those including the 3’-tag (i.e., the 3’-tagged ones, for short), and especially those including both the 3’-tag and the 5’-reverse-tag (i.e., the double-tagged ones, for short), are rare (Fig 3A).

Secondly, in the system, an important route for the appearance of parasites is from the degradation of the RNA replicase or the partial replication of the RNA replicase. In the beginning of a simulation case, raw materials are abundant, and the replication of the RNA species would be quite active. However, because of the exponential feature of the amplification, the raw materials would soon become insufficient. In fact, like any ecological systems in our modern living world, a shortage of raw materials should have been a common situation in the RNA world, which is just a key driving factor for the Darwinian evolution therein. Thus, many replicase (or its complement) molecules would stay in a “partially copied” status. Then, if the partially copied products separate from their templates, or the templates break, parasites may arise naturally. This is particularly the case for a tag-free system because there is nearly no limitation for the parasites’ sequences. Howbeit, for a tag-ruled system, parasites, especially true-parasites do not so readily appear from the “ribozyme-derived” route. In such a system, partial copying would never give rise to 3’-tagged parasites because the chain synthesis starts at 5’-end. Additionally, although the degradation may bring about 3’-tagged parasites, they would be less in quantity (than those parasites arise likewise in the tag-free system) because after the chain breaking, the 5’-end remain cannot act as a parasite. In addition, the degradation cannot result in double-tagged parasites (i.e., true-parasites in the tag-ruled system). In a test simulation, we inoculated a large number of the ribozyme molecules, and only allowed the event of RNA degradation to occur. The short RNA molecules derived from the degradation of the ribozyme molecules first increased and then decreased (Fig 3B), wherein the 3’-tagged RNA molecules adopt a same trend, but, generally, at a much lower level. Surely, the double-tagged RNA molecules cannot be found throughout the process, because only degradation is allowed–for a ribozyme to derive shorter double-tagged RNA molecules, at least two chain breakings plus a chain ligation (i.e., omitting the middle part) must occur.

Here, we should talk a little more about the true-parasites–in the context of their chain length. In the system, true-parasites are more detrimental than pseudo-parasites because they may give rise to more parasites through replication. However, long true-parasites cannot yet threaten the replicase, as already shown in Fig 2A, wherein the control species is just a long true-parasite (as long as the replicase). That is to say, one really important consequence of introducing the tag mechanism should be that “short/strong true-parasites”, instead of true-parasites per se, are difficult to appear. Obviously, in a tag-free system, either the de novo route or the ribozyme-derived route would create short true-parasites readily. In a tag-ruled system, however, things are different–short true-parasites, namely short double-tagged parasites therein, are difficult to form from random ligation (Fig 3A) or the replicase’s partial replication or degradation (Fig 3B). In fact, it is worth noting that double-tagged parasites may also appear by mutation of the ribozyme during its replication. This can also be ascribed to the ribozyme-derived route of the parasites’ emergence. Supposing that the mutation occurs in the characteristic domain of the ribozyme (the region between the two tags), a double-tagged parasite would arise and this seems not so difficult, depending on fidelity of the replication (related to *P*_*FPR*_ and *P*_*FP*_ in our model). However, the resulting true-parasite is as long as the ribozyme (similar to the control RNA species, Fig 2A), and can hardly threaten the replicase. For this long double-tagged parasite to give rise to shorter double-tagged parasites, just like the situation for the ribozyme itself (mentioned above), at least two chain breakings plus a chain ligation must occur. Certainly, there are also other ways for short double-tagged parasites to appear by chance. For example, a partially copying product (which has a 5’-reverse-tag already) happens to gain a 3’-tag by mutation and then drops from the template. But obviously, such cases should be rather occasional. Notably, in the modern living world, “replication slippage” may occur during replication and lead to the deletion of a few nucleotide residues [35,36]. Such a phenomenon, if occurring in our target system, might generate short double-tagged parasites relatively easily. However, in practice, the “replication slippage" is a rather special phenomenon. It is often associated with tandem repeats in the DNA genome, in which DNA polymerase plays a significant role. Indeed, as we know, in general, during the replication of genetic molecules, insertion-deletion mutations are much less frequent than substitution mutations. That is, no matter how, in the tag-ruled system, short/strong true parasites should be very difficult to appear.

In summary, while the tag mechanism almost does not influence the appearance of the replicase molecules, it significantly influences the appearance of parasitic molecules in the system. De novo appearance is an important way for parasitic molecules to form. But in a tag-ruled system, it is difficult for parasites to appear de novo. In particular, those short/strong true-parasites, which may generate new ones by replication and really threaten the replicase, are very difficult to appear de novo, either by random ligation or the ribozyme-derived route. This is the main reason why the tag mechanism can work and promote the prosperity of the replicase.

### Synergism between the tag mechanism and the spatial limitation mechanism

**Fig 4:**
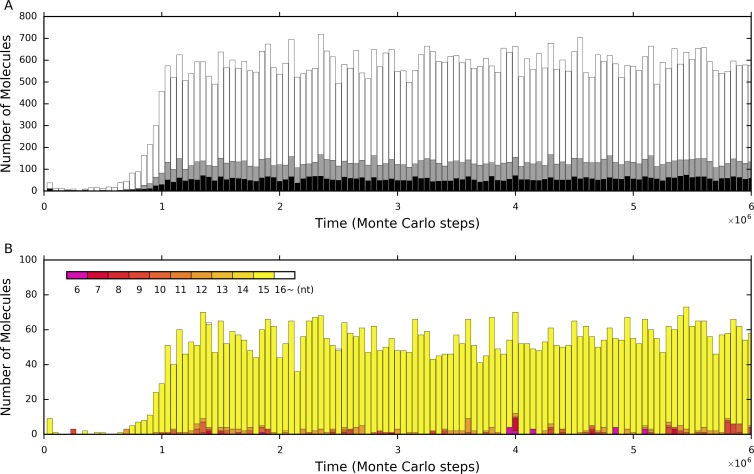
Parasites’ statistics during the spread of the replicase in the tag-ruled system. The case is the one shown in Fig 2A. (**A**) The RNA molecules other than the replicase and its complement (longer than the polymerase’s cover-length). White bars denote the molecules without the 3’-tag, gray bars denote the 3’-tagged molecules (thus, pseudo-parasites), and black bars denote the double-tagged molecules (thus, true-parasites). (**B**) The chain-length distribution of the double-tagged parasites. The longer the chain, the upper its corresponding bar is placed in the stack. The super-parasites (6nt), which have a sequence that consists of merely a 3’-tag and a 5’-retag, are denoted in purple, and the double-tagged parasites longer than the length of the double-tagged replicase (i.e., “16~”nt), are denoted in white. Others are denoted in different tones from dark red to light yellow, according to their chain-length (the longer, the lighter)–those as long as the double-tagged replicase (15nt) are in the lightest yellow. Note that those “16~” parasites are rare (e.g., see step 1.4×10^6^ and 2.5×10^6^), and this is not surprising because they are difficult to form (though not impossible, for example, by the chain breaking of two double-tagged replicases and re-ligation of the longer remains), and even if they could appear, they are at a disadvantage in the “proliferation” competition owing to their length.

Fig 4A shows the statistics of the RNA molecules which are not the replicase and its complement (but longer than the polymerase’s cover-length) during the spread of the replicase (the case of Fig 2A). Firstly, indeed, a large portion of them do not contain the 3’-tag (white bars). Thus, these RNA molecules, which would otherwise be parasites in a tag-free system, cannot act as parasites here. That is, the self-catalyzed replication of the replicase is much less interfered with in a tag-ruled system. Secondly, while we appreciate that, of all the parasites (gray + black bars), only a portion are true parasites (black bars), we somewhat wonder why there is still a substantial quantity of these double-tagged parasites (e.g., several dozen), given that they are difficult to form de novo. They are likely mutants derived from the replication of the replicase, because this is a relatively easy way (comparing with others) for double-tagged parasites to form. These long true-parasites, though not able to threaten the replicase, may still replicate and increase to some extent. Then we checked into the length distribution of the double-tagged parasites in the system (Fig 4B). The result shows that this speculation is right, because almost all the double-tagged parasites are as long as the replicase.

**Fig 5:**
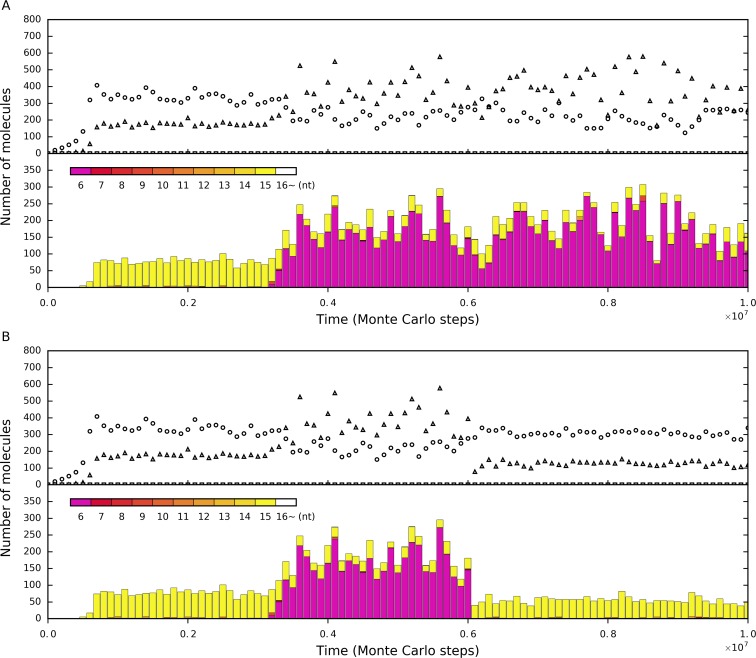
Influence of spatial limitation on the tag-ruled system. The denotations in the upper panel of the subfigures are the same as those in Fig 2A, and the denotations in the lower panel of the subfigures are the same as those in Fig 4B. (**A**) The case is that shown in Fig 2A, except that the value of *P*_*MV*_ is changed from 0.001 to 0.002. The super-parasite appears at about step 3.2 × 10^5^ and spreads in the system, thus impairing the thriving of the replicase. (**B**) When the value of *P*_*MV*_ is returned to 0.001 at step 6 × 10^5^, the super-parasite disappears and the replicase increases again.

However, we can notice that there are still some short double-tagged parasites, which show up sporadically during the whole process. That is to say, in the case shown here, although short double-tagged parasites may appear in the system occasionally, they cannot threaten the replicase also. Considering that spatial limitation has been shown to be important for early replicators to resist parasites [22,37–42], which was also evidenced in our own studies [27,30], we doubted that this mechanism may also have worked someway here, favoring the replicase. Indeed, if we adopt a higher value of *P*_*MV*_, those short double-tagged parasites, especially the shortest one–a “super-parasite” species consisting of merely a 3’-tag sequence and a 5’-reverse-tag sequence, if appearing by chance, might spread in the system and the prosperity of the replicase would be seriously compromised (Fig 5A, the parameter values are identical to those adopted in the Fig 2A case, except that *P*_*MV*_’s value is doubled). Then, if we turn down *P*_*MV*_ to the original value (as adopted in Fig 2A), the super-parasite descend and the replicase would “fight back” (Fig 5B). Surely, if, instead of turn down, we turn up *P*_*MV*_ further, the super-parasite may become more prosperous, until the replicase cannot sustain the system (S1 File). However, here, obviously, only the spatial limitation cannot take effect (we mean that the tag mechanism does work) because in a tag-free system (e.g., the cases shown in Fig 2B and 2C) we could not see any prosperity of the polymerase replicase no matter what a *P*_*MV*_ value we adopt. Taken together, these results support our speculation. That is, while the tag mechanism is important for the replicase to spread, the mechanism of spatial limitation is also important here. In particular, spatial limitation is important to suppress the spread of short true-parasites. Then, it is interesting to ask: “How could the tag mechanism cooperate with the spatial limitation mechanism in the process during which the polymerase replicase confronts parasites?”

**Fig 6:**
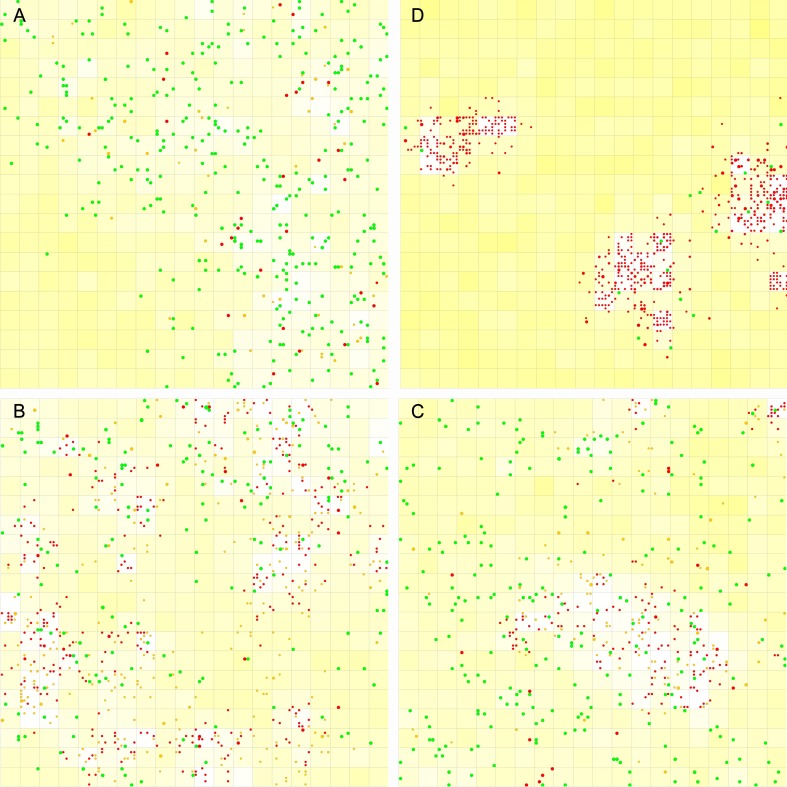
Spatial distributions of the replicase and parasites in the dynamic process. Green dots represent the double-tagged replicase (or its complement); orange dots represent the pseudo-parasites; and red dots represent the true-parasites. The length of the RNA species is represented as the diameter of the dots. For a grid room, the depth of the background yellow represents the quantity of free nucleotides therein (the deeper the color, the greater the quantity). (**A**) The case shown in Fig 2A, step 1 × 10^6^. (**B**) The case shown in Fig 5A, step 9.07 × 10^6^. (**C**) The case shown in Fig 5A, step 9.33 × 10^6^. (**D**) The case shown in Fig 2B, step 4 × 10^4^.

We traced into the spatial distributions of the replicase and the parasites in the system. In the tag-ruled system with a low *P*_*MV*_, as in Fig 2A, the polymerase spreads over the grid, while the parasites, especially the true-parasites only show up sporadically–and they tend to be long ones (as long as the double-tagged replicase; note that the sequence length is represented by the diameter of the dots) (Fig 6A). However, if a higher *P*_*MV*_ is adopted, as in Fig 5A, then when those short true-parasites, especially the super-parasites, appear by chance, they may also spread over the grid, and the prosperity of the polymerase would be obviously suppressed (Fig 6B). Significantly, the spatial distribution of the polymerase and the parasites alternates dynamically. For example, in one step (Fig 6B), the polymerase is mainly distributed in peripheral areas of the grid and the parasites are also plentiful in these areas (because there are polymerase molecules there), but at a later step (Fig 6C), the co-existing location shifts to the center of the grid, and peripheral areas become relatively “clean” areas in which the polymerase could evade parasites temporally (also see S1 Movie for a movie showing the dynamically alternating spatial distribution; the details of this phenomenon are reminiscent of the “wave dynamics” studied in the Hogeweg group, resulting from spatial limitation [37,40–42]). However, in a tag-free system, as in Fig 2B, it seems that the polymerase could never get rid of the surrounded parasites (Fig 6D).–thus, the ribozyme cannot spread in the system, even though there are still abundant building blocks (nucleotides, shown as background yellow) in other regions. What is the underlying reason that leads to these different spatial distribution structures?

**Fig 7:**
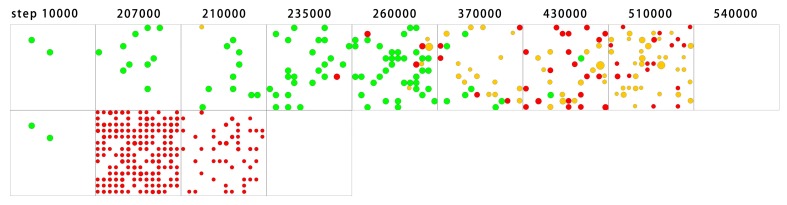
Tracking the molecular evolution in a single grid room. The denotations are the same as those in Fig 6, except that the quantity of nucleotides is not shown here (as the background yellow). The upper row shows the development of a room in a tag-ruled system. It is a room picked from a 5 × 5 grid, having the same parameter values as those used in Fig 2A, except that *P*_*MV*_ is set to 0 here. The lower row shows the development of a room in a tag-free system. It is a room picked from a 5 × 5 grid, having the same parameter values as those used in Fig 2, except that *P*_*MV*_ is set to 0 here. In each case, two molecules of the double-tagged replicase were inoculated into the room at step 1 × 10^4^, and then its development was monitored.

Firstly, the development of the replicase and the parasites in one grid room when *P*_*MV*_ is set to 0 were observed and compared in the tag-ruled and tag-free systems (Fig 7). Two molecules of the replicase were inoculated into the grid room initially and it began to proliferate. In both situations, the final state of the grid room showed the disappearance of the replicase on account of the parasitism (and the parasites also disappeared owing to the ribozyme shortage). This is understandable because the ribozyme-derived parasites are unavoidable and the replicase is an altruist (see “Introduction”). Thus, the replicase cannot get rid of the parasites when trapped in the same room, and its only chance it to thrive in the system would be to escape into other grid rooms (*P*_*MV*_ would not equal to 0 in an ordinary simulation case) before the parasites overwhelm the grid room. Here is the key point. In a tag-free system, the parasites, all of which are true-parasites and most of which are short true-parasites, are easily formed (Fig 7, the lower row, note that the sequence length is represented by the diameter of the dots), whereas in the tag-ruled system, the parasites, especially the short true-parasites, would develop much later (Fig 7, the upper row). Consequently, the tag-ruled grid room “collapses” much later than the tag-free grid room. In other words, the tag mechanism would earn more time for the replicase molecules to escape outwards into other regions and start new rounds of amplification.

Secondly, what would happen if *P*_*MV*_ is set to a large value? The influence of spatial limitation has been well elucidated by many previous studies in the area [37–42]. For the present model system, if the spatial limitation is relaxed to an extent, in which both the replicase and parasites move across the grid easier, the system would be better mixed. Then, when the replicase molecules moves to other grid rooms, they would have less chance to find a region without parasites to start a new round of amplification. Indeed, for the case shown in Fig 5A, after the spread of the super-parasite, if we turn up *P*_*MV*_ further, the super-parasite would become more prosperous, and the level of the replicase decreased further (Fig A and B in S1 File). The worst situation is that when *P*_*MV*_ reaches a rather high level, the super-parasite would pervade the whole system and the replicase could not escape from them. Thus, the whole system would collapse (Fig C in S1 File)–the replicase disappears and then the parasites disappear because of the replicase shortage. (Note: actually, the single grid room shown in Fig 7, in which the replicase and parasites are trapped together, can represent an extreme situation involving no spatial limitation, and surely, such a completely mixed system would destine to end in collapse).

In fact, the effects of spatial limitation can be observed readily in our simulations, not necessarily in regard to the super-parasite. For example, in the case shown in Fig 5B, we can see that the level of the true-parasites as long as the double-tagged replicase (yellow bars) before the spread of the super-parasite (i.e., before step 3.2 × 10^6^) is obviously higher than that (yellow bars) after we turned down the *P*_*MV*_ and the super-parasite disappeared (after step 6 × 10^6^). In fact, the only difference between the two situations is just that the *P*_*MV*_ value for the former is higher, and these long true-parasites thus have more chances to replicate in such a better mixed system. Additionally, resource depletion, as an important factor affecting the spatial dynamics, should be related to the particular sequences that are replicated. This factor may have some evolutionary consequences, for example, resulting in the coexistence of different composition-biased sequences [43]. However, for simplification, the factor is here not a focus. In particular, although our model is at the nucleotide resolution, different nucleotides are assumed to form from, and decay into, a common pool of precursors (see “Methods” for an explanation of parameters *P*_*NF*_ and *P*_*ND*_).

In summary, in a tag-ruled system, there is a synergistic effect between the tag mechanism and the spatial limitation mechanisms. While the tag mechanism would delay the replicase being overwhelmed by the parasites in a local region and thus, earn “time” for the replicase to move out and start a new round of amplification, the spatial limitation mechanism would avoid the inter-pervasion of the replicase and the parasites (especially the short true-parasites) in the system and thus, earn “space” for the replicase to move out and start a new turn of amplification. In short, this is a system with a dynamic spatial distribution that is mainly rooted in the repeated process of the replicase trying to escape from the parasites which are generated by the replicase itself. No doubt, by interpreting its synergy with the spatial limitation mechanism, we become clearer about the reason why the tag mechanism can work.

## Discussion

The origin of life on the Earth is an interesting subject. Wherein, a central issue is how Darwinian evolution could have started, given that Darwinian evolution is one of the two key aspects of the life phenomenon [44]. Darwinian evolution may have started at molecular level because molecules are natural, simple independent entities that may be subject to natural selection. The RNA replicase, which has been supposed to have initiated that hypothetic RNA world [5–8], is just such a candidate. However, the evolutionary dynamics concerning the replicate, describing how it can be self-favoring enough to catalyze its own replication, is a long-standing problem.

Here, we showed by computer simulation that the tag mechanism would be effective, but not in a manner as people previously believed. It does not work by favoring the replicase directly but by weakening the parasites in the same system. While de novo appearance is an important way for parasite molecules to occur, in a tag-ruled system, the molecules of parasites, especially those of strong parasites, have difficulty appearing de novo. In addition, the study of the cooperation between this mechanism and the spatial limitation mechanism provides us further insights into how the tag mechanism may have worked in the scenario.

The ultimate reason for the seemingly equitable tag mechanism to work against the parasites is that the added sequence limitation exerts a strong influence on the parasites, which would otherwise be completely unrestrained with regard to their sequence, while it has little influence on the replicase, which is already quite restrained in sequence because of the characteristic sequence feature of the ribozyme itself. The addition of the tag(s) to the sequence would not affect the replicase in the way it affects the parasites because almost all of the replicase molecules in the system occur owing to replication rather than de novo. Indeed, even in a tag-free system, the ribozyme is already quite restrained in sequence and we cannot “count on” that their molecules “often” appear de novo by chance. For the replicase, the negative influence of adding the tag(s), at most, means the addition of some burden to its replication, which should be a trivial factor, especially considering that in reality the ribozyme may have been much longer than the tag.

In fact, in the typical cases shown in “Results”, the shortest true-parasites in the tag-ruled system are 6nt in length (the super-parasite consisting of only a 3’-tag and a 5’-reverse-tag, both 3nt in length), whereas the shortest true-parasites in the tag-free system are 4nt in length, because the characteristic domain of the polymerase assumed in these cases is 9nt, and the cover-length of the polymerase is assumed to be (L+2)^1/2^ (note: to be bound by a polymerase, the RNA template must be longer than the cover-length of the polymerase–see “Methods”). One may argue that this is unfair to the tag-free system, and in reality this is not necessarily the case. The cover-length of the polymerase could be equal to or longer than twice of the tag’s length. We can easily exclude this factor by changing the assumption concerning the cover-length of the polymerase. For example, we can assume the cover-length to be (L+2)^1/2^+ 3, and then the shortest true-parasites in both the two systems are 7nt in length, and the results show no qualitative difference (S2 File, comparing with Fig 2). Here, the shortest true-parasites in the tag-ruled system have a sequence containing a 3’-tag, a 5’-reverse-tag and an additional nucleotide residue between them, whereas those in the tag-free system have a random sequence of 7nt. In fact, this example, in an explicit way, offers us a chance to present an alternative explanation concerning the influence of the tag mechanism. Indeed, with respect to sequence space, adding the tag(s) to the ends of the parasites’ sequences, per se, does not alter the species abundance of the parasites (see “Introduction”), but it does reduce such an abundance if the total sequence length is limited. In the present example, the sequence length is restricted to 7nt and there are only four kinds of the parasites for the tag-ruled system, which are determined by the type of the middle nucleotide residue, whereas there are 4^7^ kinds of the parasites in the corresponding tag-free system. This is a good alternative explanation especially considering that the replicase is most threatened by the shortest true-parasites. Nonetheless, this explanation is consistent with our major assertion–just because these strongest parasites are seriously limited in regard of the species abundance, they are very difficult to appear de novo, in a tag-ruled system.

In this study, two kinds of molecules were monitored throughout the simulation process, the double-tagged polymerase (as well as its complement) and the parasites, which contain a tag at their 3’-end but are not the polymerase (or its complement). In fact, another kind of molecule, the polymerase (and its complement) that contain only a 3’-tag, only a 5’-tag, or none of the tags, also deserves attention. These polymerase molecules cannot be replicated themselves, but would be functionally active. They can be deemed as “expression products” in the system. We do not focus on these molecules in this study for the sake of simplicity, but they are interesting. As it has been shown, non-replicating functional sequences, as a phenotype, may be crucial for the viability and stability of the RNA-like replicator system [45]. Perhaps we can address this aspect in a future study. In addition, it should be noted that as a portion of these expression products, polymerases (and its complement) containing a 3’-tag (but not a 5’-tag) are not counted into parasites in the present study, because parasites, as it is named, should not contribute to the molecular replication in the system. A 3’-tagged polymerase is functionally active and a 3’-tagged complement of the polymerase may direct the synthesis of a polymerase, thus contributing to the replication in the system.

To explore the effects of the tag mechanism, the characteristic domain of the replicase we designed was completely separated from the 3’-tag and the 5’-reverse-tag. Under this assumption, in principle the ribozyme benefits the RNA species with the tag(s) rather than itself only. Somewhat surprisingly, the system showed immense superiority to the tag-free system. This result indicates that the tag mechanism itself worked, nothing else. Corresponding to this assumption, a practical situation may be that the replicase recognizes the tag through some three-dimensional structural element(s), and thus the sequence of the ribozyme per se has no direct relevance with the tag. In reality, the condition may be more positive for the spread of the replicase. For example, the polymerase ribozyme might recognize the template’s 3’-tag through base-pairing with a 5’-reverse-tag contained in its own sequence. There has been experimental evidence that base-pairing was important for a polymerase ribozyme to bind onto its template [10,14–16]. If so, the complementary chain of the replicase would naturally contain a 3’-tag, and thus, the replicase would have a superior status in the tag-mediated replication.

Because the tag mechanism can work even when no superior position is given to the replicase, the mechanism may be exploited in more complex systems in which other ribozymes can also be labeled by the tag(s) and be replicated. Thus, an interesting future work is to extend the tag mechanism to RNA-based systems containing other ribozymes, like the nucleotide synthetase ribozyme and the amphiphile (membrane component) synthetase ribozyme [30–32]. Indeed, one of our original motives to study the evolutionary dynamics of the polymerase-type replicase was that it should have emerged in the RNA world sooner or later as the RNA world evolved, developing various ribozymes and thus requiring a more efficient replicase.

As mentioned already in the introduction, tag mechanism is used ubiquitously in modern organisms. Indeed, our present study suggests that the mechanism may have been important already at the very beginning of life. Once the mechanism was employed by these early life forms, we can envision that it would have become easier for them to evolve more complex mechanisms to control and regulate their own replication or metabolism because the tag(s) might readily act as handle(s) which could be exploited within these complex mechanisms. Thus, the event we study here, concerning the development of the ability to make use of tags, could be a rather significant event in early evolution, or say, in the process of the origin of life.

## Supporting information

S1 File Further relaxing the spatial limitation in the tag-ruled system. For the case shown in Fig 5A, at step 6×10^6^, PMV is turned up from 0.002 to (A) 0.01, (B) 0.05, (C) 0.5. The denotation is the same as that in Fig 5. Notably, in the case shown in C, when PMV is turned up to 0.5, the replicase and the super-parasite decrease to a level approaching zero, but they rebound some steps later, and then disappear forever. The rebound is not difficult to comprehend: due to the quick decrease of the super-parasite, some replicase molecules may gain the chance to start new turns of amplification and rise to a certain level (see C, step 6.5×10^6^), but then, on account of the “resurrection” of the replicase, the super-parasite would rebound dramatically (step 6.6×10^6^), which turns out to be the final blow for the replicase.
(TIF)

S2 File Changing the polymerase’s cover-length to equate the length of the shortest true-parasites in the tag-free system and that in the tag-ruled system. The cases shown in this figure are the same as those shown in Fig 2, except that the cover-length of the polymerase is changed from (L+2)1/2 to (L+2)1/2+3 (wherein, L represents the chain length of the characteristic domain of the ribozyme, equal to 9nt here). Note that the super-parasite in the tag-ruled system has a length of 6nt (a 3’-tag plus a 5’-reverse-tag, each 3nt), which does not exceed the polymerase’s cover-length, and thus is actually not able to act as a parasite here (to be bound by a polymerase, the RNA template must be longer than the polymerase’s cover-length–see Methods). That is, here, for either the tag-free system or the tag-ruled system, the shortest true-parasites are 7nt long.
(TIF)

S1 Movie The dynamic spatial distribution of the replicase and the parasites in the case illustrated Fig 5A (from step 9 × 10^6^ to 9.5 × 10^6^, the intervals between two frames is 1 × 10^4^ steps). See the legend of Fig 6 for the interpretation of a frame in the movie (Fig 6B and 6C are two frames from this movie).
(MP4)
