# Supplementary figures and images for: Tag mechanism as a strategy for the RNA replicase to resist parasites in the RNA world
Source: PLoS One. 2017 Mar 2;12(3):e0172702. doi: 10.1371/journal.pone.0172702 (PMC5333815; doi:10.1371/journal.pone.0172702)

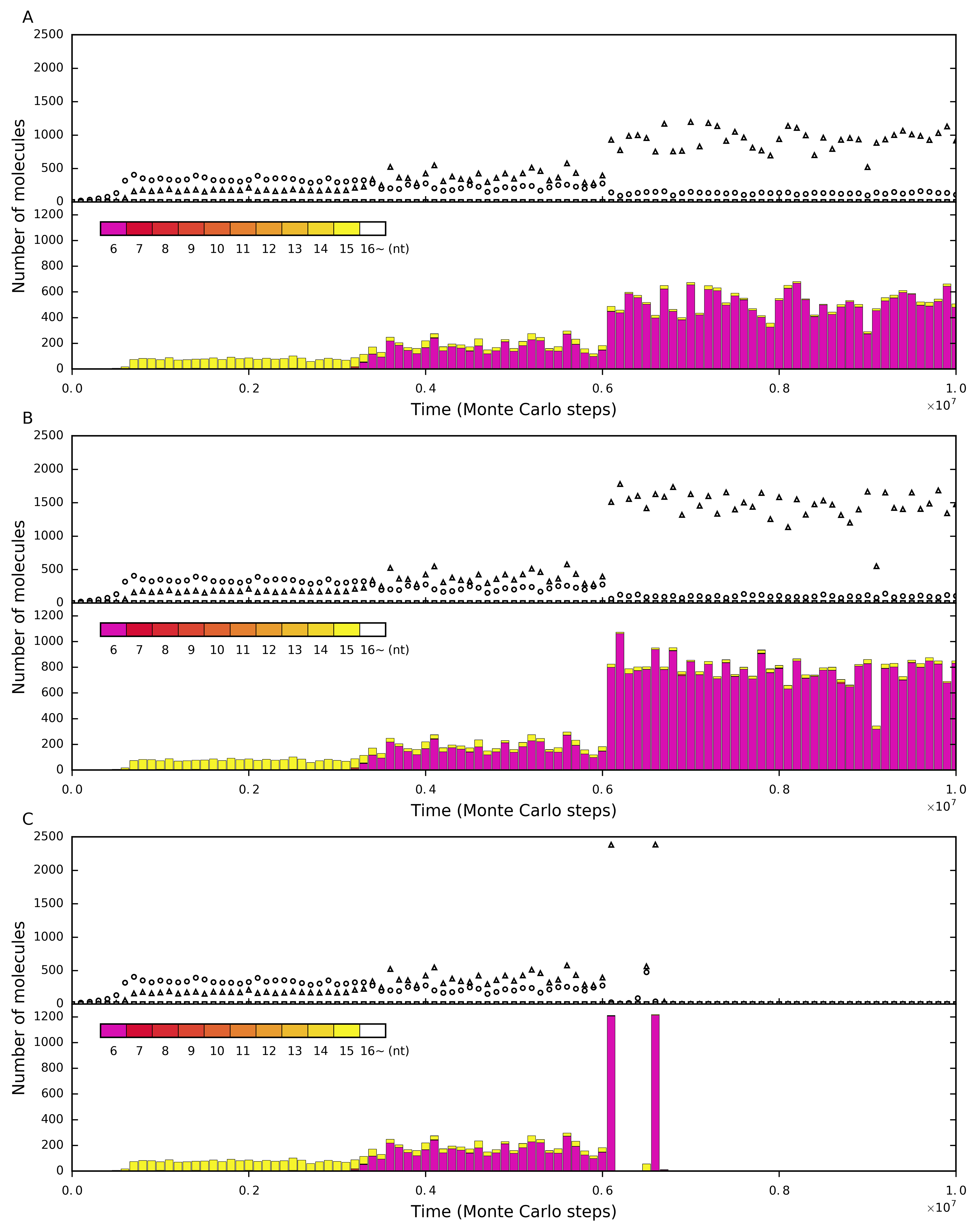

Supplement: S1 File — For the case shown in Fig 5A, at step 6×10^6^, PMV is turned up from 0.002 to (A) 0.01, (B) 0.05, (C) 0.5. The denotation is the same as that in Fig 5. Notably, in the case shown in C, when PMV is turned up to 0.5, the replicase and the super-parasite decrease to a level approaching zero, but they rebound some steps later, and then disappear forever. The rebound is not difficult to comprehend: due to the quick decrease of the super-parasite, some replicase molecules may gain the chance to start new turns of amplification and rise to a certain level (see C, step 6.5×10^6^), but then, on account of the “resurrection” of the replicase, the super-parasite would rebound dramatically (step 6.6×10^6^), which turns out to be the final blow for the replicase. (TIF) [file pone.0172702.s001.tif]

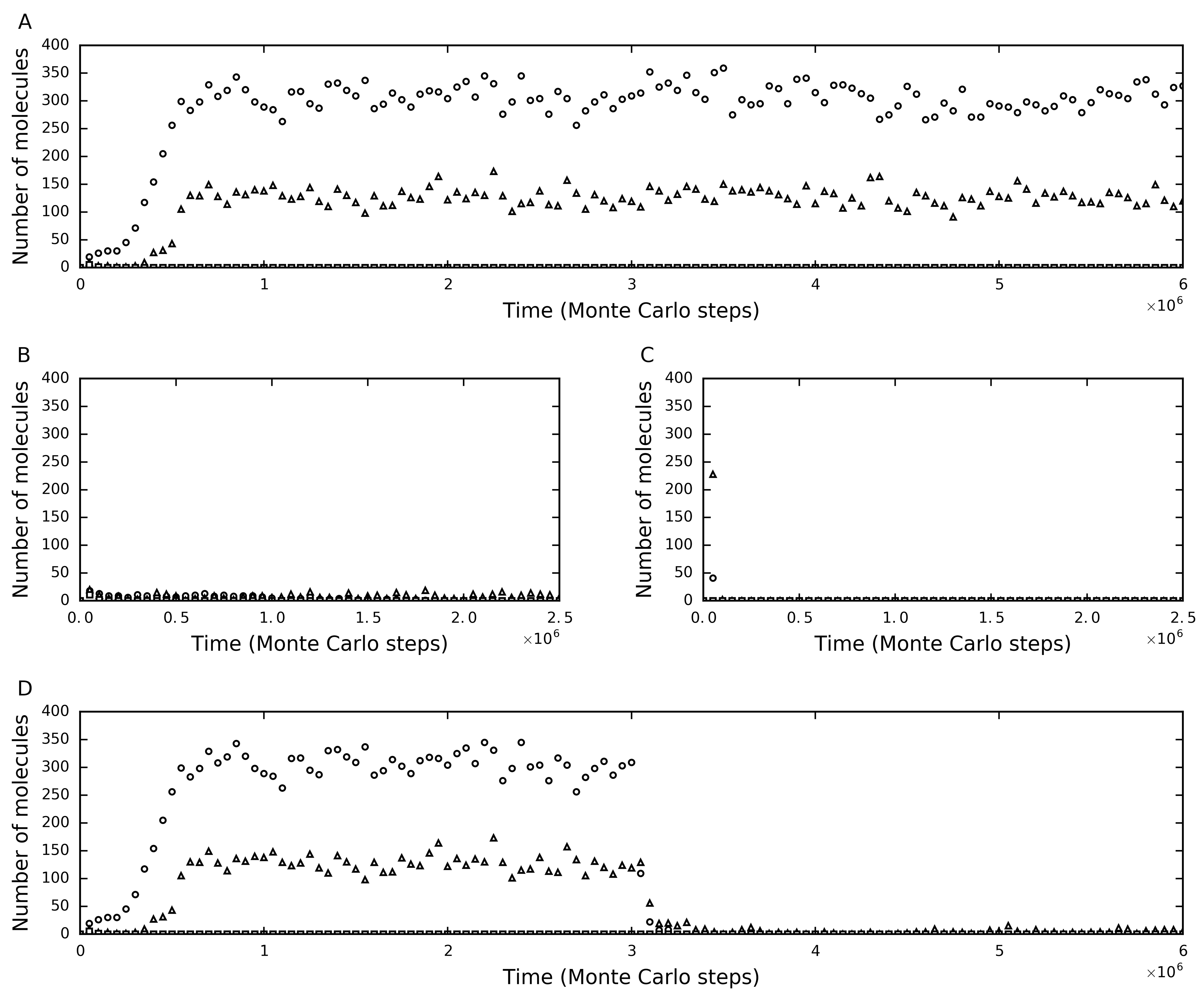

Supplement: S2 File — The cases shown in this figure are the same as those shown in Fig 2, except that the cover-length of the polymerase is changed from (L+2)1/2 to (L+2)1/2+3 (wherein, L represents the chain length of the characteristic domain of the ribozyme, equal to 9nt here). Note that the super-parasite in the tag-ruled system has a length of 6nt (a 3’-tag plus a 5’-reverse-tag, each 3nt), which does not exceed the polymerase’s cover-length, and thus is actually not able to act as a parasite here (to be bound by a polymerase, the RNA template must be longer than the polymerase’s cover-length–see Methods). That is, here, for either the tag-free system or the tag-ruled system, the shortest true-parasites are 7nt long. (TIF) [file pone.0172702.s002.tif]
